# Analysis of the use of trade names versus active ingredient names using the example of acetylsalicylic acid and Aspirin® in the *Frankfurter Allgemeine Zeitung*

**DOI:** 10.1007/s00210-025-04788-3

**Published:** 2026-01-22

**Authors:** Emma Krug, Roland Seifert

**Affiliations:** Institute of Pharmacology, Hannover Medical School, Carl-Neuberg-Str. 1, 30625 Hannover, Germany

**Keywords:** Frankfurter Allgemeine Zeitung, Acetylsalicylic acid, Aspirin®, Trade name, Active ingredient name, Drug safety

## Abstract

The *Frankfurter Allgemeine Zeitung* (F.A.Z.) is one of the most important national daily newspapers in Germany and serves as a source of information for more than 818,000 readers every day. The newspaper has high journalistic standards, which is why it should be assumed that pharmacological content is also presented correctly. Specifically, the differentiation between trade names and active ingredient names of drugs should be correct to inform readers, avoid surreptitious advertising and contribute to drug therapy safety. In this study, 345 articles from 2014 to 2023 from the online archive of the *Frankfurter Allgemeine Zeitung* were analyzed regarding the correct use of trade names using the example of Aspirin®. If the trade name is used incorrectly, suggestions are made to improve journalism about pharmacological content in the long term. The study shows that the trade name is used many times more frequently in the articles analyzed than the active ingredient name. In addition, the trade name is repeatedly mentioned in contexts where there is no need for it. This analysis shows that even in a renowned newspaper, mistakes are made in the correct naming of drugs. On this basis, further trade names and newspapers should be analyzed to counteract the "genericide" of drug names. The use of generic drug names is important for drug therapy safety.

## Introduction

The *Frankfurter Allgemeine Zeitung* (FAZ) was founded in 1949 and is based in Frankfurt am Main. It has no editor-in-chief but is managed by a board of editors and employs more than 800 people. The non-profit FAZIT Foundation has held most shares in *Frankfurter Allgemeine Zeitung* GmbH since 1959. The newspaper reaches more than 818,000 readers every day and, in addition to the print version, is also available digitally via FAZ.NET (FAZ, n.d.). The FAZ's paid circulation in the second quarter of 2024 was just under 183,000 copies (Statista 2024) and the newspaper's share of the daily newspaper market was 2.2 percent (KEK, n.d.). It is classified as conservative-liberal (Medienrot 2023) and reports freely and independently (FAZ, n.d.). The readership has an average age of 47, is well educated and has a high income. 55% of readers have a degree and the average net household income is EUR 5014 per month (Republic 2025).

As one of the highest-circulation daily newspapers in Germany, the *Frankfurter Allgemeine Zeitung* reports on many socially relevant topics. This also includes pharmacological content. One interesting aspect of the reporting is the differentiation between trade names and active ingredient names of medicines. Using the example of Aspirin®, a globally known drug developed by the Bayer Group, this paper examines the differentiation of trade and active ingredient names in the FAZ.

Since 1953, the World Health Organization has assigned a globally uniform and officially recognized International Nonproprietary Name (INN) for each active ingredient in medicinal products, which in most cases follow a specific system to enable unambiguous identification of the active ingredient (Weißer 2025). Manufacturers of new compounds apply for an INN to the responsible committee. They could suggest a name themselves. As soon as the manufacturer and the committee agree on a name, it is published. It is particularly important for a new INN that it does not resemble other names too closely and that it could not be considered inappropriate in any language. Over the years it has become apparent that new INNs are becoming longer and more complex.

Jeffrey K. Aronson summarizes the INN nomenclature system as follows: „No nomenclatural system is perfect, and the INN system is no different from any other in that respect. However, despite inconsistencies, the system has proved useful in bringing a degree of uniformity to the way in which drugs are named internationally “ (Aronson 2024).

Since the drug Aspirin® with the active ingredient acetylsalicylic acid (ASA) was developed as early as 1897, acetylsalicylic acid is not strictly speaking an International Nonproprietary Name, as the WHO classification system did not yet exist at that time. The manufacturer of the original preparation markets it under a protected brand name. This trade name is usually an invented name and often has nothing to do with the Nonproprietary Name (Graefe et al. 2016). The trade name is identified by an ®. After patent protection expires, other pharmaceutical manufacturers may also produce and market the product. These products are referred to as generics (Federal Ministry of Health, 2024). Some generic drug manufacturers also use brand names, further complicating the situation for consumers and medical professionals.

In addition to its analgesic, anti-inflammatory and antipyretic effects, ASA also has an antiplatelet effect (Schrör, 2011). Acetylsalicylic acid is suitable for secondary prophylaxis of ischemic cardiovascular events (Cooper and Skinner 2009).

The effect of acetylsalicylic acid is based on the irreversible acetylation of cyclooxygenase. This results in an irreversible inhibition of prostaglandin synthesis and reduced thromboxane A2 formation (Seifert 2018). The patenting of Aspirin® was rejected by Germany in 1899. It was successfully patented in the USA and the UK (DPMA, 2025), but these patents have also long since expired. Nevertheless, the trade name Aspirin®, which was granted by Bayer, is now so firmly associated with the active ingredient name acetylsalicylic acid that many people no longer use the name as a brand name, but as a synonym for the active ingredient itself.

The Bayer Group is active worldwide in the fields of health care and agriculture (Bayer 2025). It was founded in 1863 by Friedrich Bayer and Johann Friedrich Weskott. In 1881, the Group was transformed into a stock corporation (Bayer 2024) and in 2018 the US seed and herbicide manufacturer Monsanto was acquired (Bayer 2018).

Using the example of Aspirin®, this paper aims to investigate whether the differentiation of trade and active ingredient names in a renowned print medium such as the *Frankfurter Allgemeine Zeitung* is correct, as it serves as a source of information for a large readership and therefore also bears a special responsibility in drug reporting. To the best of our knowledge, there is no comparable work in Germany, so this study can serve as a basis for further analyses of other journals and trade names.

## Material and methods

**Fig. 1:**
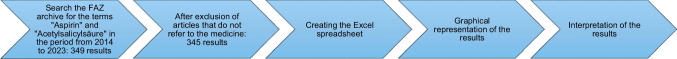
Schematic representation of the analysis

The procedure during the analysis is shown in Fig. 1. After identifying the topics, a period from 2014 to 2023 was selected. In addition, the search terms “Aspirin” and “Acetylsalicylsäure” were defined to search the online archive of the *Frankfurter Allgemeine Zeitung* for suitable articles (F.A.Z.-Bibliotheksportal, n.d.). Free access to the archive was made possible by the *Niedersächsische Landesbibliothek*. Within the specified period, 349 articles were published by the F.A.Z. containing at least one of the two search terms. After excluding articles that did not use “Aspirin” to refer to the drug, a total of 345 articles were analyzed. An Excel table was then created, which summarizes individual articles and assigns them contexts and suggestions for improvement that were previously defined. Tables 1, 2, and 3 show the classification system of the content of articles related to “Aspirin”. Table 4 shows an extract from the raw data table. Once all the articles had been analyzed, the results were presented graphically and interpreted in relation to the topic of the paper.

**Table 1:** Classification of the contexts in which the trade name was used within an article

Number	Meaning
1	Use of the trade name in connection with the Bayer Group
2	The trade name becomes clear through the context as a brand without mentioning the Bayer Group
3.1	The trade name is used within a direct Quotation without mentioning the Bayer Group
3.2	The trade name is used within an indirect Quotation without mentioning the Bayer Group
4	The trade name is used in a medical context and context categories 1–3 do not apply
5	The trade name is not used in any medical context and context categories 1–3 do not apply

**Table 2:** Classification of improvement suggestions for the articles

Letter	Meaning
a	No improvement required as only the active ingredient name was mentioned
b	No improvement required, because correct use of the trade name and identification as a registered trademark by the corresponding symbol ®
c	Mention of the trade name is appropriate in the context, but should nevertheless be identified as a registered trademark by the associated symbol ®
d	When using the trade name and the name of the active ingredient, there should be sufficient differentiation between the two designations, and they should be identified as a registered trademark by the associated ® symbol
e	The active ingredient name should be used instead of the trade name
f	The trade name should be replaced by a more general formulation such as "tablet", as neither the trade name nor the name of the active ingredient is relevant to the content of the article at this point

**Table 3:** Classification of the differentiation between trade name and active ingredient name, if both designations are used within an article

Letter	Meaning
A	When the trade name and the active substance name are mentioned, sufficient differentiation is made between the two designations, and the meaning of the active ingredient name is made clear
B	When the trade name and the active ingredient name are mentioned, there is insufficient differentiation between the two designations. The trade name is used as a synonym for the active ingredient name.

**Table 4:** Extract of the raw data of the analyzed articles

Article	Word Count	Department	Frequency of active ingredient name	Frequency trade name	Tagged ®	Context of naming trade name (systematized)	Context of naming trade name and active ingredient name (systematized)	Suggestion for improvement (systematized)
1	682	no information	0	3	No	4	no information	e
2	1822	Sunday newspaper	0	1	No	1	no information	c
3	915	no information	0	2	No	3.1	no information	c
4	915	Sport	0	1	No	3.1	no information	c
5	1678	Sunday newspaper	0	2	No	1	no information	c
6	1791	no information	0	2	No	1	no information	c
7	353	Economy	0	1	No	1	no information	c
8	681	Economy	0	1	No	1	no information	c
9	668	FAZ.NET	0	1	No	1	no information	c
10	529	Rhine-Main Newspaper	2	3	No	3.2	B	d
11	546	FAZ.NET	2	3	No	3.2	B	d
12	543	no information	2	3	No	3.2	B	d
13	803	Pictures and times	0	1	No	5	no information	f
14	991	Economy	0	1	No	1	no information	c
15	998	no information	0	1	No	1	no information	c
16	453	Economy	0	1	No	1	no information	c
17	425	FAZ.NET	0	1	No	1	no information	c
18	1891	Politics	0	1	No	3.1	no information	c
19	1903	no information	0	1	No	3.1	no information	c
20	519	FAZ.NET	0	1	No	1	no information	c
21	559	Sunday newspaper	0	1	No	1	no information	c
22	204	Nature and Science	1	3	No	4	B	d
23	308	Economy	0	1	No	1	no information	c
24	934	Economy	0	1	No	1	no information	c
25	883	FAZ.NET	0	1	No	1	no information	c
26	262	FAZ.NET	0	1	No	2	no information	c
27	335	FAZ.NET	0	2	No	5	no information	f
28	355	FAZ.NET	0	1	No	4	no information	c
29	312	Profession and Opportunity	0	1	No	5	no information	f
30	368	Nature and Science	0	1	No	3.1	no information	c
31	348	FAZ.NET	0	1	No	3.1	no information	c
32	1070	Economy	0	1	No	2	no information	c
33	2072	FAZ.NET	0	1	No	3.1	no information	c
34	2129	Sunday newspaper	0	1	No	3.1	no information	c
35	1047	Nature and Science	0	1	No	4	no information	e
36	1029	FAZ.NET	0	1	No	4	no information	e
37	2282	FAZ.NET	0	1	No	3.2	no information	c
38	2299	no information	0	1	No	3.2	no information	c
39	2276	Sunday newspaper	0	1	No	3.2	no information	c
40	1346	Profession and Opportunity	0	1	No	4	no information	f
41	1311	FAZ.NET	0	1	No	4	no information	f
42	951	Economy	0	1	No	5	no information	f
43	915	FAZ.NET	0	1	No	5	no information	f
44	1256	Sunday newspaper	1	1	No	5	A	c
45	1138	Feuilleton	0	1	No	5	no information	f
46	803	FAZ.NET	0	1	No	4	no information	e
47	2104	FAZ.NET	0	1	No	3.2	no information	c
48	2131	Sunday newspaper	0	1	No	3.2	no information	c
49	445	FAZ.NET	0	1	No	1	no information	c
50	432	Rhine-Main Newspaper	0	1	No	1	no information	c
51	620	Rhine-Main Newspaper	0	1	No	3.1	no information	c
52	658	Magazine	0	1	No	4	no information	f
53	273	Economy	0	1	No	2	no information	c
54	2262	Magazine	0	1	No	3.1	no information	c
55	2783	Feuilleton	0	1	No	3.1	no information	c
56	873	FAZ.NET	0	1	No	2	no information	c
57	2702	FAZ.NET	0	1	No	3.1	no information	c
58	622	Politics	0	1	No	4	no information	e
59	631	FAZ.NET	0	1	No	4	no information	e
60	932	Enterprise	0	1	No	2	no information	c
61	785	Economy	0	1	No	1	no information	c
62	750	FAZ.NET	0	1	No	1	no information	c
63	1403	Economy	0	1	No	1	no information	c
64	1327	FAZ.NET	0	1	No	1	no information	c
65	1465	FAZ.NET	0	1	No	1	no information	c
66	1689	Sunday newspaper	0	1	No	1	no information	c
67	2049	Sunday newspaper	0	1	No	4	no information	e
68	2002	FAZ.NET	0	1	No	4	no information	e
69	4549	no information	0	1	No	3.1	no information	c
70	316	FAZ.NET	0	1	No	3.2	no information	c
71	3363	Sunday newspaper	1	1	No	2	A	c
72	3360	FAZ.NET	1	1	No	2	A	c
73	3408	no information	1	1	No	2	A	c
74	414	Economy	0	1	No	3.1	no information	c
75	900	Sunday newspaper	0	1	No	2	no information	c
76	1038	Publisher's supplement	0	1	No	4	no information	e
77	1188	Sunday newspaper	1	1	No	3.2	A	c
78	1159	FAZ.NET	1	1	No	3.2	A	c
79	585	Sunday newspaper	0	1	No	3.2	no information	c
80	546	FAZ.NET	0	1	No	3.2	no information	c
81	782	FAZ.NET	0	1	No	3.2	no information	c
82	1096	FAZ.NET	0	1	No	3.2	no information	c
83	1106	Rhine-Main Newspaper	0	1	No	3.2	no information	c
84	413	Sport	0	2	No	3.2	no information	c
85	385	FAZ.NET	0	2	No	3.2	no information	c
86	662	Politics	0	2	No	4	no information	e
87	653	FAZ.NET	0	2	No	4	no information	e
88	1627	Sunday newspaper	0	1	No	1	no information	c
89	1595	FAZ.NET	0	1	No	1	no information	c
90	1351	Sunday newspaper	0	1	No	5	no information	f
91	4550	no information	0	1	No	3.1	no information	c
92	8574	no information	0	1	No	3.1	no information	c
93	734	FAZ.NET	0	1	No	5	no information	f
94	952	FAZ.NET	0	1	No	4	no information	f
95	1002	Rhine-Main Newspaper	0	1	No	4	no information	f
96	1312	Economy	0	1	No	5	no information	f
97	1317	FAZ.NET	0	1	No	5	no information	f
98	3200	Quarterly	0	1	No	5	no information	f
99	1550	Politics	0	1	No	3.1	no information	c
100	1495	FAZ.NET	0	1	No	3.1	no information	c
101	774	Feuilleton	0	1	No	2	no information	c
102	732	FAZ.NET	0	1	No	2	no information	c
103	1056	FAZ.NET	0	1	No	4	no information	e
104	1204	Knowledge	0	1	No	4	no information	e
105	1050	Economy	1	1	No	2	A	c
106	1139	Publisher's supplement	0	1	No	4	no information	e
107	1318	Nature and Science	1	1	No	2	A	c
108	1234	FAZ.NET	1	1	No	2	A	c
109	1909	FAZ.NET	0	1	No	5	no information	f
110	2068	Sunday newspaper	0	1	No	5	no information	f
111	1280	Sunday newspaper	0	1	No	1	no information	c
112	816	Rhine-Main Newspaper	0	1	No	3.1	no information	c
113	755	FAZ.NET	0	1	No	3.1	no information	c
114	233	Economy	0	1	No	3.1	no information	c
115	607	no information	0	1	No	3.1	no information	c
116	378	Rhine-Main Newspaper	0	1	No	3.1	no information	c
117	3680	no information	0	1	No	3.1	no information	c
118	1035	FAZ.NET	0	1	No	3.1	no information	c
119	10,208	no information	0	1	No	1	no information	c
120	597	FAZ.NET	0	1	No	1	no information	c
121	363	Economy	0	1	No	1	no information	c
122	300	FAZ.NET	0	1	No	1	no information	c
123	1047	FAZ.NET	0	2	No	3.1	no information	c
124	1231	Sunday newspaper	0	1	No	5	no information	e
125	1160	FAZ.NET	0	1	No	5	no information	e
126	2426	Travel sheet	0	1	No	5	no information	e
127	2259	FAZ.NET	0	1	No	5	no information	e
128	2191	Sunday newspaper	0	1	No	4	no information	f
129	2146	FAZ.NET	0	1	No	4	no information	f
130	486	FAZ.NET	0	1	No	5	no information	e
131	520	Sunday newspaper	0	1	No	5	no information	e
132	660	Rhine-Main Newspaper	0	1	No	5	no information	f
133	1089	Sunday newspaper	0	4	No	1	no information	c
134	920	FAZ.NET	0	3	No	1	no information	c
135	666	Economy	0	1	No	1	no information	c
136	460	Economy	0	1	No	1	no information	c
137	1401	Publisher's supplement	0	1	No	4	no information	e
138	989	FAZ.NET	0	4	No	1	no information	c
139	1053	Rhine-Main Newspaper	0	4	No	1	no information	c
140	356	Rhine-Main Newspaper	0	1	No	5	no information	f
141	333	FAZ.NET	0	1	No	5	no information	f
142	877	FAZ.NET	1	1	No	1	B	d
143	1918	Sunday newspaper	0	1	No	4	no information	f
144	580	Economy	0	1	No	1	no information	c
145	1058	Economy	0	1	No	1	no information	c
146	972	no information	0	1	No	1	no information	c
147	830	Economy	0	1	No	1	no information	c
148	1265	Nature and Science	0	1	No	3.2	no information	c
149	1214	FAZ.NET	0	1	No	3.2	no information	c
150	386	FAZ.NET	0	1	No	4	no information	e
151	2869	Sport	0	1	No	3.1	no information	c
152	2794	FAZ.NET	0	1	No	3.1	no information	c
153	496	Feuilleton	0	1	No	4	no information	e
154	464	FAZ.NET	0	1	No	4	no information	e
155	1153	FAZ.NET	1	2	No	3.1	A	c
156	1711	Sunday newspaper	0	2	No	3.2	no information	c
157	1628	FAZ.NET	0	2	No	3.2	no information	c
158	949	Sunday newspaper	0	1	No	2	no information	c
159	875	FAZ.NET	0	1	No	2	no information	c
160	171	FAZ.NET	0	3	No	1	no information	c
161	935	Economy	0	1	No	3.1	no information	c
162	1853	Feuilleton	0	1	No	3.1	no information	c
163	883	FAZ.NET	0	2	No	3.1	no information	c
164	1807	FAZ.NET	0	1	No	3.1	no information	c
165	1252	Economy	0	1	No	3.2	no information	c
166	392	Sunday newspaper	0	1	No	2	no information	c
167	5115	Sunday newspaper	0	2	No	1	no information	c
168	1074	Nature and Science	0	2	No	3.2	no information	c
169	997	FAZ.NET	0	2	No	3.2	no information	c
170	1152	Sunday newspaper	0	1	No	5	no information	f
171	1658	FAZ.NET	0	3	No	3.1	no information	c
172	1688	Sunday newspaper	0	2	No	3.1	no information	c
173	445	Economy	0	1	No	1	no information	c
174	227	Economy	0	2	No	1	no information	c
175	1522	Sunday newspaper	0	1	No	5	no information	f
176	1486	FAZ.NET	0	1	No	5	no information	f
177	1137	Quarterly	0	3	No	3.1	no information	c
178	661	Sunday newspaper	0	1	No	3.1	no information	c
179	194	Economy	0	1	No	1	no information	c
180	602	Sunday newspaper	0	1	No	4	no information	f
181	287	FAZ.NET	0	1	No	2	no information	c
182	674	FAZ.NET	0	1	No	3.2	no information	c
183	304	Economy	0	8	No	1	no information	c
184	452	Rhine-Main Newspaper	0	1	No	5	no information	f
185	759	Economy	0	1	No	1	no information	c
186	868	Editorial supplement	0	1	No	4	no information	e
187	670	Feuilleton	0	1	No	5	no information	f
188	591	Economy	0	1	No	1	no information	c
189	524	FAZ.NET	0	1	No	1	no information	c
190	887	Rhine-Main Newspaper	0	2	No	2	no information	c
191	850	Society	0	1	No	4	no information	e
192	1276	Literature supplement	0	1	No	3.2	no information	c
193	1185	Sport	0	1	No	3.1	no information	c
194	841	Economy	0	1	No	5	no information	f
195	2756	Nature and Science	2	2	No	4	B	d
196	2576	FAZ.NET	2	2	No	4	B	d
197	933	Economy	0	1	No	5	no information	f
198	1667	Editorial supplement	0	1	No	3.2	no information	c
199	1549	FAZ.NET	0	1	No	3.2	no information	c
200	1060	Economy	0	1	No	1	no information	c
201	729	Rhine-Main Newspaper	0	1	No	4	no information	f
202	1690	Economy	0	2	No	1	no information	c
203	2158	Sunday newspaper	0	1	No	4	no information	e
204	427	FAZ.NET	0	1	No	4	no information	f
205	280	Economy	0	1	No	1	no information	c
206	460	Profession and Opportunity	0	1	No	4	no information	f
207	892	Economy	0	1	No	1	no information	c
208	2023	Economy	0	1	No	3.2	no information	c
209	622	Economy	0	1	No	1	no information	c
210	934	Economy	0	1	No	3.2	no information	c
211	857	FAZ.NET	0	1	No	3.2	no information	c
212	923	Publisher's supplement	0	1	No	4	no information	e
213	693	Economy	0	1	No	1	no information	c
214	608	FAZ.NET	0	1	No	1	no information	c
215	1173	Sunday newspaper	0	1	No	4	no information	e
216	1155	FAZ.NET	0	1	No	4	no information	e
217	677	Economy	0	1	No	1	no information	c
218	2628	Sunday newspaper	0	2	No	1	no information	c
219	341	FAZ.NET	0	1	No	3.2	no information	c
220	583	Rhine-Main Newspaper	0	1	No	2	no information	c
221	1628	Sport	0	1	No	3.1	no information	c
222	1503	FAZ.NET	0	1	No	3.1	no information	c
223	1238	Economy	0	1	No	1	no information	c
224	830	Economy	0	1	No	5	no information	e
225	729	FAZ.NET	0	1	No	5	no information	e
226	539	Economy	0	1	No	1	no information	c
227	1242	Economy	0	1	No	3.2	no information	c
228	1239	Sunday newspaper	0	1	No	4	no information	e
229	1198	FAZ.NET	0	1	No	4	no information	e
230	1161	Economy	0	1	No	1	no information	c
231	1447	Sunday newspaper	0	1	No	1	no information	c
232	1014	Politics	0	1	No	1	no information	c
233	944	FAZ.NET	0	1	No	1	no information	c
234	1311	Economy	0	1	No	3.2	no information	c
235	549	FAZ.NET	0	2	No	3.1	no information	c
236	625	FAZ.NET	0	1	No	3.1	no information	c
237	1345	FAZ.NET	0	1	No	5	no information	f
238	1508	Sunday newspaper	0	1	No	5	no information	f
239	984	Feuilleton	0	1	No	3.1	no information	c
240	1195	FAZ.NET	0	1	No	3.2	no information	c
241	1253	Sunday newspaper	0	1	No	3.2	no information	c
242	2169	Sunday newspaper	0	1	No	3.2	no information	c
243	2130	FAZ.NET	0	1	No	3.2	no information	c
244	281	FAZ.NET	0	1	No	3.2	no information	c
245	316	Sunday newspaper	0	1	No	3.2	no information	c
246	1488	Sunday newspaper	0	1	No	3.1	no information	c
247	1054	Economy	0	1	No	1	no information	c
248	844	Economy	0	2	No	1	no information	c
249	1771	FAZ.NET	0	1	No	5	no information	f
250	650	Rhine-Main Newspaper	0	1	No	5	no information	f
251	591	FAZ.NET	0	1	No	5	no information	f
252	2479	Sunday newspaper	0	1	No	2	no information	c
253	2055	FAZ.NET	0	1	No	2	no information	c
254	889	Sunday newspaper	0	2	No	1	no information	c
255	846	FAZ.NET	0	2	No	1	no information	c
256	783	Rhine-Main Newspaper	0	1	No	3.1	no information	c
257	1170	Economy	0	1	No	2	no information	c
258	1112	FAZ.NET	0	1	No	2	no information	c
259	2087	Sunday newspaper	0	1	No	3.2	no information	c
260	827	Economy	0	1	No	1	no information	c
261	1006	Economy	0	1	No	5	no information	f
262	879	Economy	0	1	No	1	no information	c
263	751	FAZ.NET	0	1	No	1	no information	c
264	2285	Sunday newspaper	0	1	No	5	no information	f
265	720	Rhine-Main Newspaper	0	1	No	3.2	no information	c
266	1937	FAZ.NET	0	1	No	5	no information	f
267	1362	FAZ.NET	0	1	No	5	no information	f
268	1381	Sunday newspaper	0	1	No	5	no information	f
269	2523	FAZ.NET	0	1	No	5	no information	f
270	1815	Magazine	0	1	No	5	no information	f
271	2561	Magazine	0	1	No	5	no information	f
272	212	Politics	0	1	No	3.1	no information	c
273	520	Economy	0	1	No	1	no information	c
274	845	Economy	0	1	No	5	no information	f
275	793	FAZ.NET	0	1	No	5	no information	f
276	1072	Economy	0	1	No	2	no information	c
277	1101	Economy	0	1	No	2	no information	c
278	1010	Economy	0	1	No	2	no information	c
279	809	Economy	0	1	No	2	no information	c
280	439	Sunday newspaper	0	1	No	5	no information	f
281	1104	Economy	0	1	No	2	no information	c
282	1063	Economy	0	1	No	2	no information	c
283	986	Economy	0	1	No	2	no information	c
284	394	Feuilleton	0	1	No	3.2	no information	c
285	351	FAZ.NET	0	1	No	3.2	no information	c
286	1164	Economy	0	1	No	2	no information	c
287	1110	Economy	1	21	No	1	A	c
288	1101	Economy	0	2	No	2	no information	c
289	1268	FAZ.NET	0	1	No	3.2	no information	c
290	1312	Profession and Opportunity	0	1	No	3.2	no information	c
291	1083	Economy	0	1	No	1	no information	c
292	2023	Travel sheet	0	1	No	5	no information	f
293	1974	FAZ.NET	0	1	No	5	no information	f
294	542	Sunday newspaper	0	2	No	3.1	no information	c
295	497	FAZ.NET	0	2	No	3.1	no information	c
296	1328	Sunday newspaper	1	23	No	1	A	c
297	1260	FAZ.NET	1	23	No	1	A	c
298	2557	Sunday newspaper	0	1	No	4	no information	e
299	2490	FAZ.NET	0	1	No	4	no information	e
300	614	FAZ.NET	0	1	No	1	no information	c
301	962	Sunday newspaper	1	1	No	4	B	d
302	823	FAZ.NET	1	1	No	4	B	d
303	803	Sunday newspaper	0	1	No	3.2	no information	c
304	944	Youth writes	0	2	No	4	no information	e
305	701	Sunday newspaper	0	1	No	4	no information	e
306	1183	Rhine-Main Newspaper	0	1	No	5	no information	f
307	2117	Sunday newspaper	0	1	No	3.1	no information	c
308	2035	FAZ.NET	0	1	No	3.1	no information	c
309	685	Feuilleton	2	1	No	4	B	d
310	962	Sunday newspaper	0	1	No	3.1	no information	c
311	750	FAZ.NET	0	1	No	3.1	no information	c
312	389	Rhine-Main Newspaper	0	2	No	5	no information	f
313	304	Economy	0	1	No	1	no information	c
314	1023	Economy	0	1	No	1	no information	c
315	339	Sunday newspaper	0	5	No	3.1	no information	c
316	400	FAZ.NET	0	5	No	3.1	no information	c
317	421	Economy	0	1	No	1	no information	c
318	644	Economy	0	1	No	1	no information	c
319	410	Rhine-Main Newspaper	0	1	No	5	no information	f
320	823	Sunday newspaper	0	1	No	5	no information	f
321	775	Economy	0	3	No	1	no information	c
322	1697	Travel sheet	0	1	No	3.1	no information	c
323	260	Economy	0	1	No	1	no information	c
324	409	Economy	0	1	No	1	no information	c
325	205	Politics	0	1	No	4	no information	f
326	253	Economy	0	1	No	1	no information	c
327	850	Economy	0	1	No	1	no information	c
328	505	Sunday newspaper	0	1	No	3.1	no information	c
329	696	FAZ.NET	0	1	No	5	no information	e
330	795	Economy	0	1	No	5	no information	e
331	1496	FAZ.NET	1	0	no information	no information	no information	a
332	1602	Sunday newspaper	1	0	no information	no information	no information	a
333	1152	no information	1	0	no information	no information	no information	a
334	1133	Publisher's supplement	1	0	no information	no information	no information	a
335	4024	no information	1	0	no information	no information	no information	a
336	568	Economy	2	0	no information	no information	no information	a
337	1023	Sunday newspaper	1	0	no information	no information	no information	a
338	1625	Sunday newspaper	1	0	no information	no information	no information	a
339	1604	FAZ.NET	1	0	no information	no information	no information	a
340	138	Rhine-Main Newspaper	1	0	no information	no information	no information	a
341	569	Nature and Science	1	0	no information	no information	no information	a
342	137	Rhine-Main Newspaper	1	0	no information	no information	no information	a
343	812	Nature and Science	1	0	no information	no information	no information	a
344	770	FAZ.NET	1	0	no information	no information	no information	a
345	253	FAZ.NET	1	0	no information	no information	no information	a

## Results

### Frequency of mentions of the trade name

**Fig. 2:**
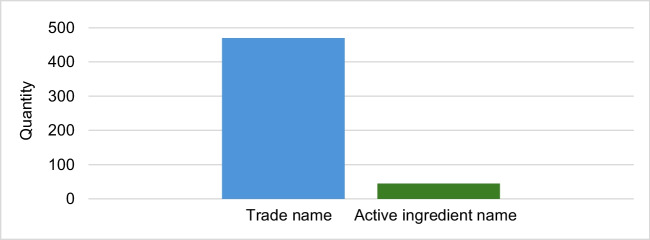
Number of mentions of the trade name and the active ingredient name

**Fig. 3:**
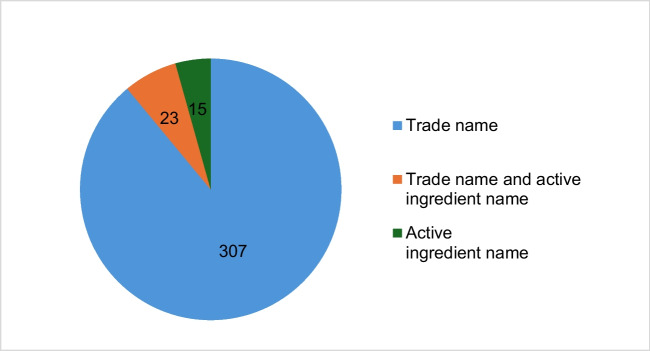
Number of articles in which only the trade name, only the active ingredient name and both be mentioned together

Figure 2 shows the total number of mentions of the trade name and the active ingredient name in the articles analyzed. The trade name is mentioned much more frequently than the active ingredient name. The trade name Aspirin® is mentioned a total of 470 times from 2014 to 2023 inclusive, while the active ingredient name is only mentioned 45 times in the same period (see Fig. 2). As can be seen in Fig. 3, only the trade name is mentioned in 307 of the 345 articles, without any additional mention of the active ingredient name. In a further 23 articles, both the trade name and the active ingredient name are mentioned, and 15 articles contain only the active ingredient name.

**Fig. 4:**
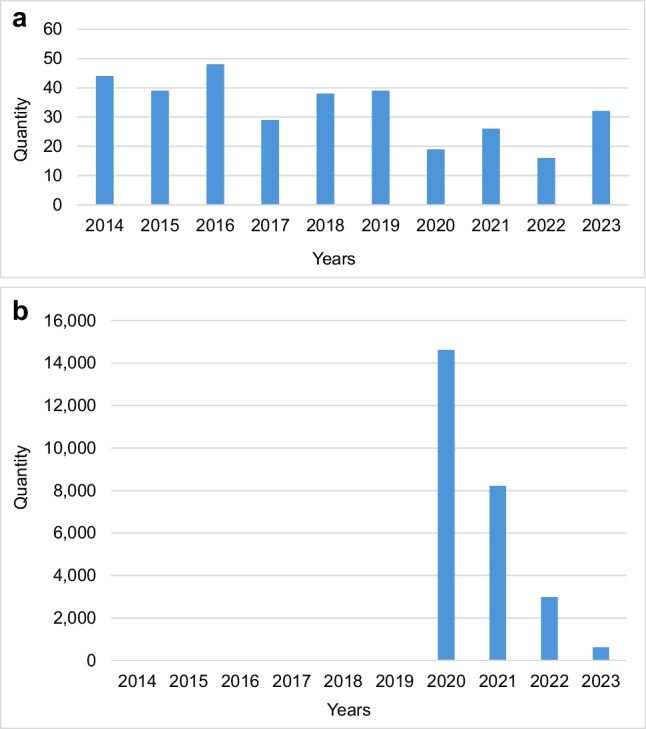
**a** Number of articles in which the trade name is mentioned, from 2014 to 2023 **(b)** Number of articles published under the search term "Coronavirus" from 2014 to 2023

The number of articles mentioning the trade name shows a downward trend between 2020 and 2022 (see Fig. 4a). At the same time, the number of articles containing the search term "Coronavirus" in the FAZ archive increased dramatically, as shown in Fig. 4b.

**Fig. 5:**
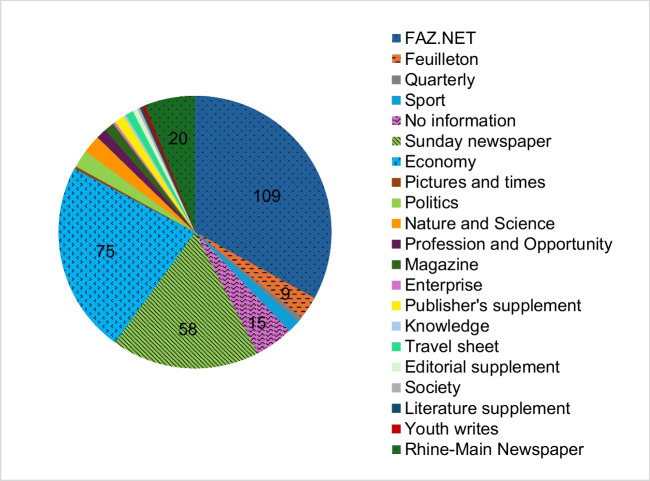
Number of articles in which the trade name is mentioned in the various departments

Figure 5 shows that the trade name is used particularly frequently in articles in the FAZ.NET, Economy and Sunday Newspaper sections. In none of the articles is the trade name identified as a registered trademark.

### Classification of the use of the trade name in contexts

To assess the necessity of using the trade name Aspirin® in a specific context, the numbers 1 to 5 are assigned to different context categories (see Table 1). Context 1 is assigned if the trade name is used in connection with the Bayer Group. If it is clear from the context that the trade name is a brand name without mentioning the Bayer Group, the article is assigned to context 2. Context 3 indicates the use of the trade name within a quotation also without mentioning the Bayer Group, whereby context 3.1 describes a direct quotation and context 3.2 an indirect quotation. If none of contexts 1 to 3 apply, but the trade name is mentioned in a medical context, context 4 is assigned. If neither context 1 to 3 nor a medical reference is present, context 5 is assigned. If the trade name is used more than once within an article, the article is assigned the context that is most appropriate overall.

**Fig. 6:**
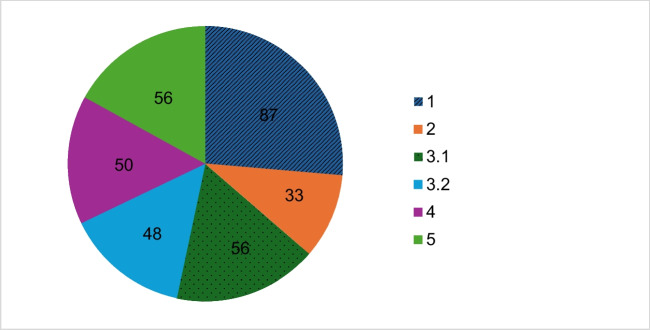
Number of all articles divided into contexts in which the trade name is mentioned

Figure 6 shows that the trade name is mentioned in 87 of the 345 articles and thus most frequently in context 1.

**Fig. 7:**
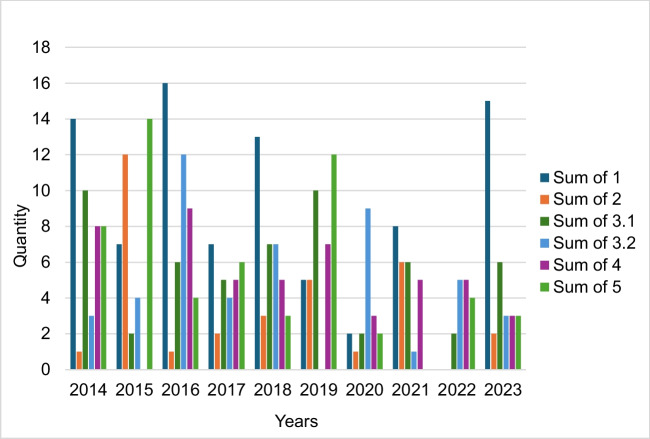
Number of contexts in which the trade name is mentioned, from 2014 to 2023

When looking at the temporal course of the frequency of the assigned contexts in Fig. 7, it is noticeable that the trade name was mentioned particularly often in the years 2014, 2016, 2018 and 2023 in connection with the Bayer Group (context 1).

### Assignment of suggestions for improvement

To contribute to improved journalism, each article is assigned a suggestion for improvement, which is marked with a letter from a to f (see Table 3). An a is assigned if no improvement is required, as only the active ingredient name appears in the respective article. If the trade name is used correctly, including the identification as a registered trademark, b is assigned. If the trade name is used appropriately in the relevant context, but is not identified as a registered trademark, the improvement suggestion c is assigned, as this identification should always take place. If both the trade name and the active ingredient name are mentioned within an article, but without clearly differentiating between the two designations, improvement suggestion d contains a clear differentiation and the identification of the trade name as a registered trademark. If the active ingredient name is sufficient in the context of the article and can therefore replace the trade name, the improvement proposal is assigned to e. If neither the trade name or the active ingredient name is important in the context of the article, but more general formulations such as “pain medication” or “tablet” would be sufficient, suggestion f is assigned.

**Fig. 8:**
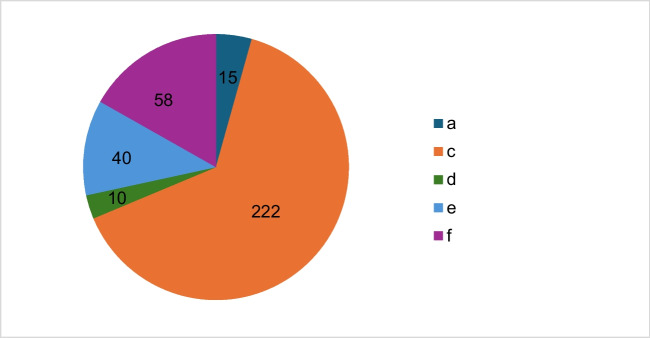
Number of all articles divided into suggestions for improvement

Figure 8 shows that in 222 articles the identification as a registered trademark is missing, but the trade name is used correctly in the article. However, in 58 articles both the trade name and the active ingredient name are superfluous and in 40 articles it is recommended to replace the trade name with the active ingredient name. Improvement suggestion b, which indicates the correct use of the trade name including the identification as a registered trademark, could not be awarded, as this identification did not occur in any of the articles analyzed.

**Fig. 9:**
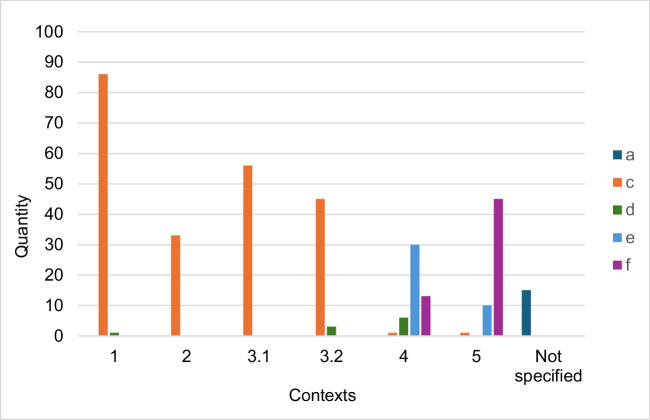
Number of suggestions for improvement within the contexts

Figure 9 shows which suggestions for improvement can be assigned to the respective contexts in which the trade name is mentioned. Various suggestions for improvement can be assigned to contexts 4 and 5. For articles that could be assigned to contexts 1 to 3, the improvement suggestion c was predominantly assigned, because in these contexts the use of the trade name Aspirin® is generally appropriate, but the labeling as a registered trademark should still be used. All articles in which only the active ingredient name is used and therefore no context was assigned are summarized under “not specified” and assigned to improvement suggestion a.

### Differentiation between trade and active ingredient names

In 23 of the articles analyzed, both the trade name and the active ingredient name were mentioned. A categorization is made to assess the selectivity between the two terms. A clear differentiation between trade name and active ingredient name is labeled A, while an unclear differentiation or the use of the terms as synonyms is labeled B (see Table 2).

**Fig. 10:**
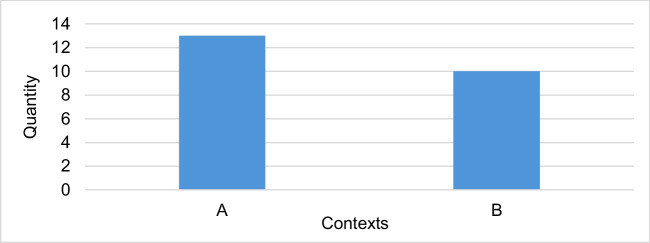
Number of contexts in articles that mention the trade name and the active ingredient name

Although most articles make a clear distinction between the terms, Fig. 10 shows that in ten articles the exact difference between the two terms is not clearly emphasized despite the mention of both the trade name and the active ingredient name.

### Number of words

**Fig. 11:**
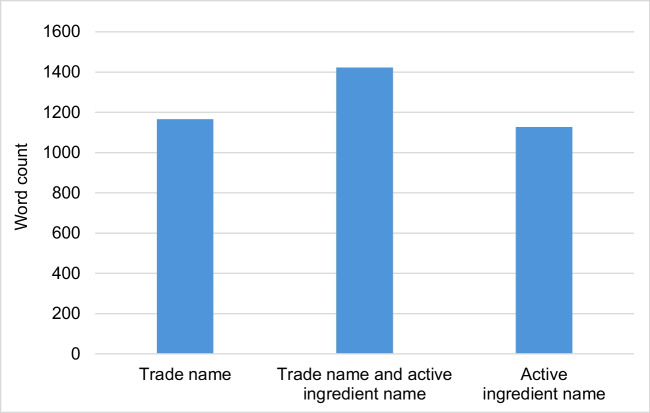
Mean values of the number of words in the articles in which the trade name, the active ingredient name, and the trade name and the active ingredient name are mentioned

Figure 11 shows the mean values of the number of words in the articles in which only the trade name, only the active ingredient name, as well as the trade name and the active ingredient name are mentioned. In articles in which both the trade name and the active ingredient name are mentioned, the average word count is over 200 words higher than in articles containing only one of the terms.

## Discussion

The analysis of the results shows that the trade name Aspirin® still has a high status in reporting despite the existence of many generics. The trade name is also used in articles that report neither on the Bayer Group or on medical issues.

**Fig. 12:**
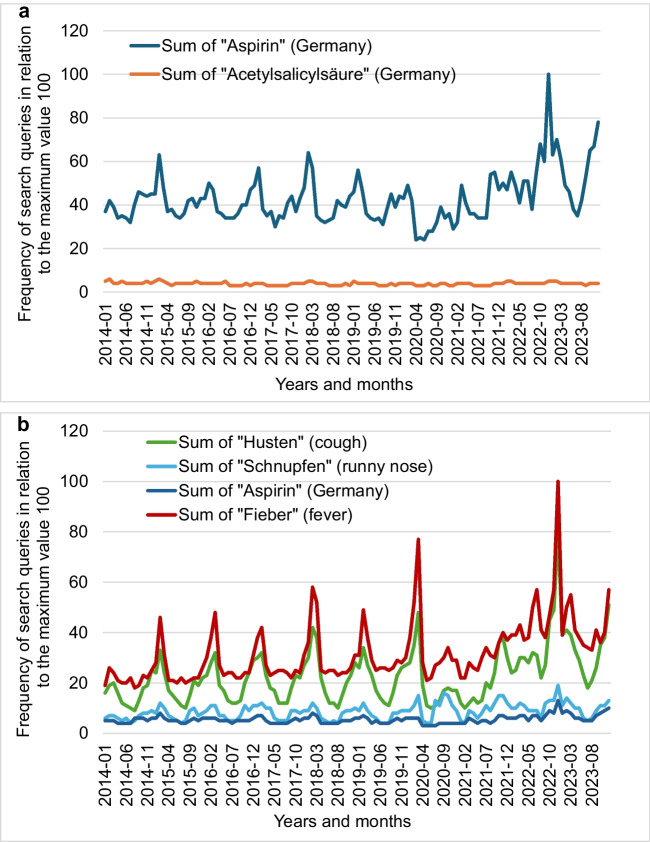
**a** Frequency of Google searches for "Aspirin" and "Acetylsalicylsäure" from 2014 to 2023 in Germany. (**b)** Frequency of Google searches for "Aspirin", „Husten" (cough), „Schnupfen “ (runny nose) and „Fieber “ (fever) from 2014 to 2023 in Germany

Figure 12a looks at the search queries on Google for “Aspirin” and "Acetylsalicylsäure" (acetylsalicylic acid) from 2014 to 2023 in relation to a maximum value defined as 100. Search queries for “Aspirin” are significantly more frequent than search queries for “Acetylsalicylsäure” (Google Trends 2024). The "Aspirin" curve also reflects the seasonal variability of respiratory infections, as the number of search queries increased significantly, especially in the winter months. The increase in search queries after 2020 could also be related to the coronavirus pandemic and the associated sensitivity to respiratory diseases, as these were the focus of political and media attention during this time. Particularly striking in this context is the peak in search queries from October 2022 to March 2023. It is reasonable to assume that this increase is due to an increase in respiratory diseases after the period of isolation. This assumption is supported, as shown in Fig. 12b, by the simultaneous increase in search queries for "Husten" (cough), "Schnupfen" (runny nose) and " Fieber" (fever) as these terms were also searched for more frequently during this period (Google Trends 2025).

Comparing Figs. 4a and 4b, it becomes clear that the decline in mentions of the trade name between 2020 and 2022 could be related to the dominant reporting on the global coronavirus pandemic.

To find out whether the awareness of the trade name is related to the prescription figures, the results are compared with the Drug Prescription Report. The Drug Prescription Report records the frequency of drug prescriptions via statutory health insurance. Based on the prescription figures for 2022, the report shows that Aspirin® from the Bayer Group plays a rather subordinate role compared to generic preparations. In 2022, 6.2 million daily doses of Aspirin® were prescribed. This corresponds to a loss of 13.7% compared to the previous year. In contrast, 693.3 million daily doses of acetylsalicylic acid from other manufacturers were prescribed. This means that Aspirin® only accounts for 0.89% of prescribed acetylsalicylic acid preparations in 2022. The average cost of the defined daily dose for Aspirin® is 12 cents, which is three to four times higher than the cost of generics (Ludwig et al. 2023).

This comparison shows that there is a large discrepancy between the awareness of the term Aspirin® and its actual significance in terms of prescriptions on the German pharmaceutical market. There are many possible reasons for this awareness. On the one hand, not every journalist is probably aware of the difference between the trade name and the active ingredient name of a medicinal product. On the other hand, trade names are usually shorter and catchier than the active ingredient names and are therefore easier for the public to understand. Another reason for the familiarity of Aspirin® may be the Bayer Group's decades-long investment in advertising for the product. As a result, the brand name has become deeply anchored in the collective memory and is passed on from generation to generation. Over time, the trade name became part of common usage, and its original character as a trade name was lost. This phenomenon is referred to as "genericide" and affects other well-known brand names (Cornell Law School 2022). The "genericide" of Aspirin® may also have been due to the fact that Aspirin® is no longer a protected brand name in the USA, among other countries, because the brand name was confiscated in several countries during the First World War (German Patent and Trade Mark Office, 2025). The FAZ may have been influenced by English-language scientific literature. A combination of these factors favors the mutual reinforcement between the media presence and the awareness of the trade name among the population.

When evaluating the contexts in which the trade name is mentioned, it became clear that context 1, which mentions in connection with the Bayer Group, was particularly prevalent in 2014, 2016, 2018 and 2023. To find possible causes for this, the history of the Bayer Group was examined more closely. It is noticeable that a lot of attention was focused on the Group in these years for various reasons. In March 2014, Bayer took over the Algeta Group, which specializes in cancer therapy. In 2016, the takeover of seed manufacturer Monsanto is planned, and the takeover is successfully completed in 2018 (Bayer 2023). The Bayer Group reports losses of around €2.9 billion for 2023 (Tagesschau 2024). Such reports and the resulting increased attention on the Group may be the reason for the frequent mentions of Aspirin® in connection with the Bayer Group in the years mentioned.

Our study also show that the trade name was never identified as a registered trademark during the period under investigation. Such labeling would help readers to clearly identify the trade name as a trademark, which would lead to less confusion with the active ingredient name.

The lack of a registered trademark could give the impression that the trade name is used as a general term for painkillers, which could unintentionally create a certain advertising effect for the product. By contrast, the correct labeling of trade names as registered trademarks would maintain journalistic neutrality and a clear distinction between trade name and active ingredient name would also be possible for medical laypersons.

The clear differentiation of trade and active ingredient names in reporting plays a central role in drug therapy safety. The use of active ingredient names or International Nonproprietary Names facilitates communication about medicinal products, both between different professional groups and between different nationalities. Correct differentiation of the two designations can also protect against overdoses by enabling patients and medical staff to recognize different preparations with identical active ingredients as such. If, for example, only the trade name is noted on the patient's medication plan, there is a risk that patients will take another preparation with the identical active ingredient and thus exceed the maximum recommended daily dose.

Another problem with the use of the trade name is the risk of confusion between medicinal products and other active ingredients due to similar-sounding trade names, as unlike the International Nonproprietary Names, these are not subject to a system. There is also a risk with over-the-counter preparations that patients will buy the more expensive original product in the belief that only this can help them. In addition, in the event of supply bottlenecks, the lack of knowledge about active ingredient names can lead to patients not knowing that the same active ingredient is available from another manufacturer.

Overall, the analysis shows that the *Frankfurter Allgemeine Zeitung* already practices an appropriate and differentiated use of trade and active ingredient names in many places in the articles examined. There is potential for optimization in the consistent identification of trade names as registered trademarks and the use of the active ingredient name in articles in which the trade name and the naming of a trademark offer no added value for the article. The inaccuracies observed regarding the correct differentiation between trade and active ingredient names probably do not indicate a deliberate preference for certain brands but are rather due to a lack of awareness among journalists of the relevance of this distinction. It is essential that everyone reporting on drugs is familiar with the drug name system and uses it correctly. Distinguishing between trade names and INNs in pharmaceutical reporting forms the basis for responsible journalism.

Against this background, targeted training for journalists could help them to understand the importance of precise terminology in a pharmacological context and thus promote even more neutral reporting and make an important contribution to drug safety in Germany.

### Limitations

Our analysis is based on a limited sample of 345 articles, which limits the generalizability of the results. In addition, only one newspaper was examined, which means that no general conclusions can be drawn about journalism. The study also focuses exclusively on the use of one specific trade name. This limits the informative value in relation to other brands. The methodology used is also based on a subjective interpretation of the language, which potentially limits the objectivity of the categorization. Podcast transcripts were also included in the analysis to ensure the completeness of the search results in the FAZ archive in the defined period. An incorrect transcription, in which “Aspirin” was transcribed but not pronounced, led to the exclusion of this article from the analysis. In addition, not every mention of “Aspirin” necessarily refers to the drug. For example, articles in which “Aspirin” was used to refer to a type of rose were also not included in the analysis.

A further limitation of this work is the analysis of the online archive, which is a dynamic medium. Content can be added, deleted or changed. Furthermore, some of the articles analyzed are almost identical in terms of content, as they appeared in different sources (e.g. *Frankfurter Allgemeine Zeitung* and FAZ.NET) and therefore appear several times in the archive.

Due to the inaccessibility of sales figures for over-the-counter Aspirin®, it was not possible to include these figures in the discussion section of the paper. For this reason, reference can only be made to the known data from the Drug Prescription Report on prescription Aspirin®.

### Future studies

Future work could contribute to a more accurate picture of the use of trade names in journalism by analyzing other brands. In addition, it would be interesting to integrate other media formats into the analysis to gain a more comprehensive view of the role of trade names in current media. In addition, looking at a longer period could reveal possible changes in the use of trade names over the decades.

## Data Availability

All data from the project are available from the authors upon reasonable request.

## Abbreviations

ASA: Acetylsalicylic acid
DPMA: German Patent and Trademark Office
FAZ: Frankfurter Allgemeine Zeitung
INN: International Nonproprietary Name
KEK: Commission on Concentration in the Media
